# Microscopic Mechanism of Asphalt Mixture Reinforced by Polyurethane and Silane Coupling Agent: A Molecular Dynamics Simulation-Based Study

**DOI:** 10.3390/polym17121602

**Published:** 2025-06-09

**Authors:** Zhi Lin, Weiping Sima, Xi’an Gao, Yu Liu, Jin Li

**Affiliations:** School of Civil Engineering, Sichuan University of Science & Engineering, Zigong 643000, China; linzhi@suse.edu.cn (Z.L.); simaweiping@suse.edu.cn (W.S.); gaoxian@suse.edu.cn (X.G.); liuyu@suse.edu.cn (Y.L.)

**Keywords:** asphalt mixture, molecular simulation, polyurethane modifier, warm mixing temperature, silane coupling agent

## Abstract

Most modified asphalts require high-temperature shearing and prolonged mixing to achieve a uniform structure, often resulting in substantial exhaust gas pollution. This study explores the utilization of polyurethane (PU) as a warm mix asphalt modifier, leveraging its favorable compatibility with asphalt at lower temperatures to mitigate emissions. To address the inherent limitations of PU-modified asphalt mixtures, namely, poor low-temperature performance and susceptibility to water damage, silane coupling agents (SCAs) are introduced to reinforce the asphalt–aggregate interfacial strength. At the microscopic level, the optimal PU content (20.8%) was determined through analysis of micro-viscosity and radial distribution functions (RDFs). SCA effects on interfacial properties were assessed using adhesion work, adhesion depth, and interfacial thermal stability. At the macroscopic level, performance metrics—including strength, high-temperature resistance, low-temperature resistance, and water stability—were evaluated against a benchmark hot mix SBS-modified asphalt mixture. The results indicate that PU-modified asphalts exhibit superior high-temperature performance and strength but slightly lower low-temperature performance and insufficient water stability. The addition of SCAs improved both low-temperature and water stability attributes, enabling the mixtures to meet specification requirements. The simulation results suggest that KH-550, which chemically reacts with isocyanate groups (-OCN) in PU, exhibits a better interfacial reinforcement effect than KH-570. Therefore, KH-550 is recommended as the preferred SCA for PU-modified asphalt mixtures in practical applications.

## 1. Introduction

With the construction and development of asphalt pavement, the problems of smoke emission and asphalt aging in hot mix asphalt mixtures have gradually attracted people’s attention [1,2,3]. Harmful gas emissions not only impose a certain burden on the environment but more importantly, they pose risks and harm to the health of road construction workers [4]. According to relevant studies, the lowering of the mixing temperature can effectively reduce the emission of harmful gases such as smoke, CO, NOx, and SO_2_ and improve the service life of the equipment [5,6,7]. A common method to reduce the mixing temperature of asphalt mixtures is by adding warm mix additives (such as synthetic waxes) to improve asphalt fluidity, ensuring better workability under warm mix conditions. This facilitates easier compaction and enhances pavement density. However, such additives often negatively impact the road performance of asphalt mixtures—for instance, increasing brittleness at low temperatures, raising the risk of cracking, and posing uncertainties regarding long-term durability [8,9]. Therefore, it is an urgent problem to find an asphalt modifier that can reduce mixing temperature but can achieve the properties of hot mix asphalt mixtures. PU has good compatibility with asphalt; the best mixing temperature is within the temperature range of warm mix asphalt. The PU-modified asphalt mixture has excellent road performance, and many scholars have carried out research on this [10]. Li Z compared and analyzed the microstructures of PU-modified asphalt and SBS-modified asphalt by means of infrared spectroscopy (IR) and found that the compatibility between PU and asphalt is better than that of SBS [10]. Jia M used the SBS-modified asphalt mixture as the control group to verify the performance of thermosetting polyurethane (TS-PU) and the thermoplastic polyurethane (TP-PU)-modified asphalt mixture, and both of them have good high-temperature performance, fatigue resistance, and strength performance [11].

The incorporation of polyurethane (PU) into asphalt generally improves not only its high-temperature stability and low-temperature crack resistance but also enhances fatigue resistance and durability. However, some studies have identified water susceptibility issues in PU-modified asphalt mixtures. This is primarily attributed to PU’s inherent hydrophilicity—the polar groups in PU tend to form weak bonds with asphalt surfaces that are easily disrupted by water molecules, consequently reducing the mixture’s moisture-induced stripping resistance. Many scholars fill in the lack of poor water stability by increasing the content of PU or preparing composite PU modifiers, but these two methods both improve the overall properties of the mixture by improving the performance of the adhesive material and lack the pertinence to the water damage resistance of the material [10,12,13]. Therefore, from the perspective of enhancing the asphalt–aggregate interface, this paper uses SCAs to improve the interface performance of the PU-modified asphalt mixture under warm mixing temperatures [14,15]. The SCA is a surface treatment agent with many types. In recent years, its application in the interface enhancement of road materials has gradually increased [16,17]. Yu J uses SCAs to strengthen the rubber–cement interface, and the results show that SCAs can effectively repair the interface defects between the two [18]. Xiang Y used an electron microscope to investigate the interface performance of asphalt–rubber powder after adding SCAs. It was found that the SCA significantly improved the absorption performance of rubber powder to asphalt and enhanced overall strength and resistance to deformation [17].

**Figure 1:**
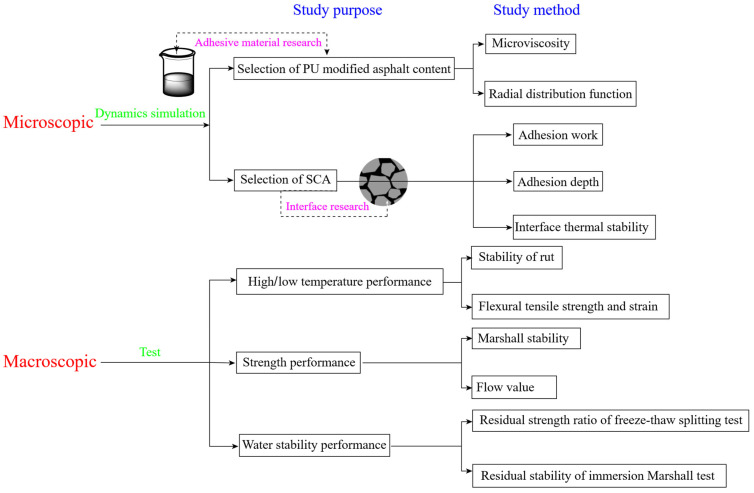
Research roadmap of this study.

Both PU and SCAs are industrial materials with many categories and certain activities. If you want to select PU and SCAs suitable for asphalt and aggregate reinforcement, it is necessary to explore the microscopic mechanism of the interaction between several materials. Molecular dynamics simulation is a material research method and has made great progress in the research of asphalt modifiers in recent years [19,20,21]. This paper will employ molecular dynamics theory to first conduct a comparative analysis of different PU modifier models to determine the optimal PU modifier content. Then, by investigating the performance enhancement differences of various SCA molecular models on the asphalt–aggregate interface, we will further explore the influence of SCAs on interface enhancement from a microscopic perspective, identifying the molecular types that improve asphalt–aggregate interface performance and predicting the SCA molecules that can best react with the isocyanate groups in PU modifiers. Finally, through macroscopic fundamental experiments using hot mix SBS-modified asphalt mixtures as the control group, we will evaluate the performance of warm mix PU-modified asphalt mixtures and investigate the performance improvement effects of PU-modified asphalt mixtures with added SCAs. The specific parameter indicators and research process are shown in Figure 1.

## 2. Materials and Methods

### 2.1. Materials

**Figure 2:**
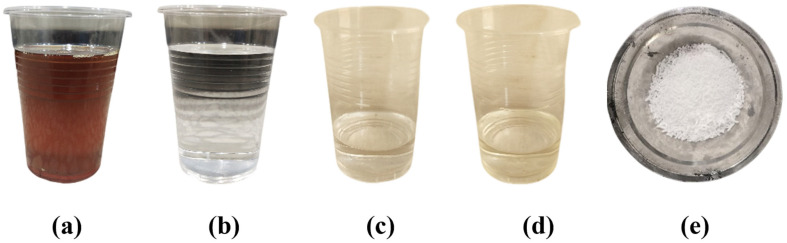
Materials utilized in this study: (**a**) PU; (**b**) BDO; (**c**) KH-550; (**d**) KH-570; (**e**) SBS.

The asphalt selected in this paper is CNOOC 90# asphalt, as shown in Table 1. The origin of the asphalt is Taizhou, China and the producer is China National Offshore Oil Corporation (CNOOC). The main component of PU is Wanhua PM200 polymerized isocyanate, as shown in Table 2 and Figure 2a. 1,4-Butanediol (BDO) was selected as the chain extender, as shown in Table 3 and Figure 2b. The SCAs selected are KH-550 and KH-570, as shown in Table 4 and Figure 2c,d. SBS is a linear thermoplastic styrene–butadiene rubber, as shown in Table 5 and Figure 2e. The origin of the modifier is Huizhou, China, and the manufacturer is Huizhou Li Changrong Rubber Co.

### 2.2. Molecular Dynamics Simulation

Molecular dynamics simulation is a microscopic simulation method based on Newton’s second law, which has a wide range of applications in the field of materials science. The principle is to calculate a series of parameters, such as the velocity and acceleration of molecules, through Newton’s equation of motion (Equation (1)) and Newton’s law of motion equation (Equations (2)–(4)).

(1) $${\vec{a}}_{i}=\frac{{\vec{F}}_{i}}{m_{i}}$$

(2) $$\frac{d^{2}}{dt^{2}}{\vec{r}}_{i}=\frac{d}{dt}{\vec{v}}_{i}={\vec{a}}_{i}$$

(3) $${\vec{v}}_{i}={\vec{v}}_{i}^{0}+{\vec{a}}_{i}t$$

(4) $${\vec{r}}_{i}={\vec{r}}_{i}^{0}+{\vec{v}}_{i}^{0}t+\frac{1}{2}{\vec{a}}_{i}t^{2}$$

where F, m, r, v, t, and a represent the force, mass, position, velocity, time, and acceleration of the molecule, respectively, and i represents the molecular serial number.

### 2.3. Micro Model Establishment

#### 2.3.1. Asphalt Molecular Model

**Figure 3:**
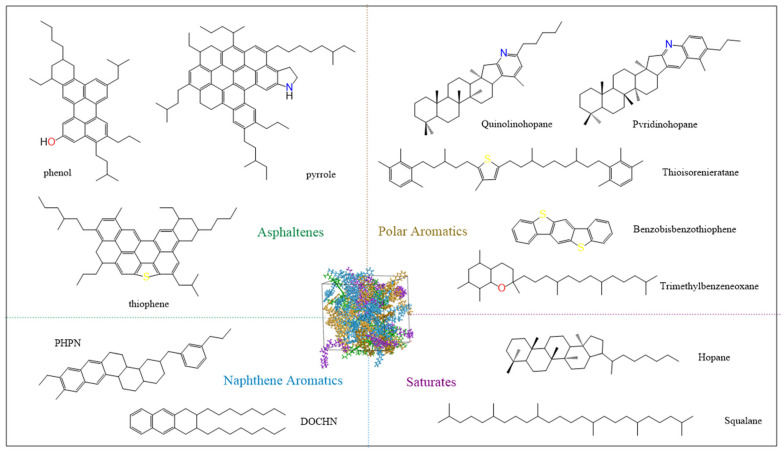
Molecular structure of asphalt with four components.

Asphalt is a complex mixture composed of various hydrocarbons and their derivatives. Li and Greenfield established molecular models for 12 components of AAA-1 asphalt by analyzing variations in molecular structures, elemental compositions, and solubility parameters, thereby correlating macroscopic parameters with molecular characteristics [22,23]. In this study, we adopt the four-component twelve-molecule model proposed by Li to construct specific molecular structures representing asphaltenes, resins, aromatic phenols, and saturated phenols. The characteristic molecular names are shown in Figure 3, with each molecular model built using the drawing tools in Materials Studio 2019.

#### 2.3.2. PU Molecular Model

**Figure 4:**
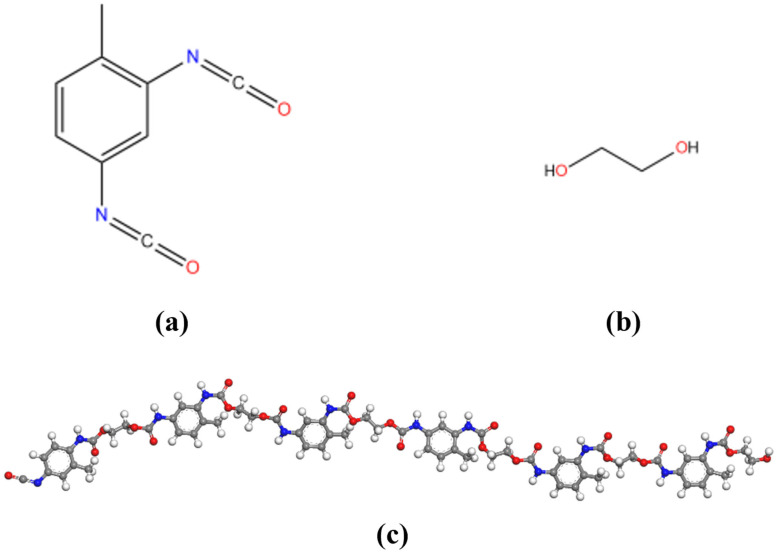
The molecular structure: (**a**) 2,4 TDI; (**b**) EG; (**c**) PU.

Polyurethane (PU) is widely used in various industries due to its excellent elastic deformation capability, low thermal conductivity, and superior bonding properties, and its outstanding performance in asphalt modification makes it an optimal modifier material. As a high-molecular-weight organic polymer, PU consists of repeating structural units formed through the interaction of hard segments, soft segments, and hinge structures. In this study, we employ polyurethane (PU) synthesized from the reaction between hydroxyl groups on polyols (with the highest degree of polymerization) and isocyanate groups on isocyanates to produce long-chain polymer molecules containing carbamate groups (-NHCOO-). To ensure the accuracy of molecular dynamics calculations, the degree of polymerization (DP) is set to 6, where the isocyanate component is 2,4-toluene diisocyanate (TDI) and the polyol component is ethylene glycol [24]. The specific molecular structures of these components are shown in Figure 4a,b, respectively, and the final polyurethane molecular structure is presented in Figure 4c.

#### 2.3.3. Establishment and Verification of Asphalt and the PU-Modified Asphalt Model

**Figure 5:**
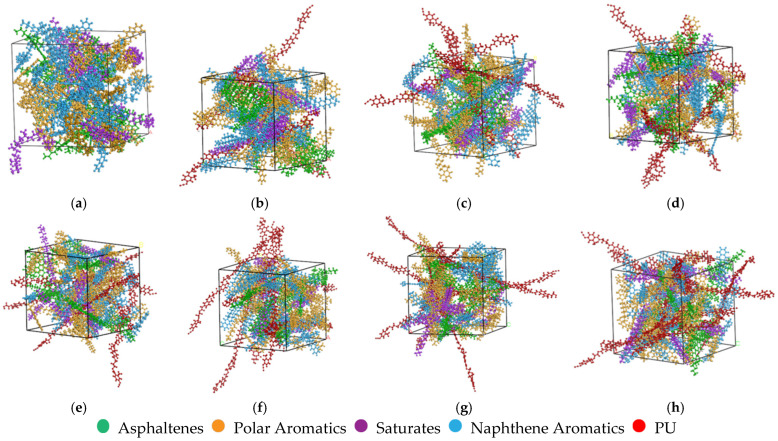
Models of asphalt and PU-modified asphalt: (**a**) Matrix asphalt; (**b**) 11.6% PU; (**c**) 14.9% PU; (**d**) 17.9% PU; (**e**) 20.8% PU; (**f**) 23.4% PU; (**g**) 25.9% PU; (**h**) 28.2% PU.

The four components ratio is obtained through the component analysis test, and the asphalt molecular model is assembled according to its requirements. The specific number of molecules and the proportion of each component are shown in Table 6. The final matrix asphalt model is shown in Figure 5a. Adding PU molecules to the existing matrix asphalt model, the number of PU molecules is 3-9, respectively, and its proportion in the PU-modified asphalt is 11.6%, 14.9%, 17.9%, 20.8%, 23.4%, 25.9%%, and 28.2% respectively. The specific models are shown in Figure 5b–h.

The solubility parameter is a physical index that measures the degree of compatibility between different polymers, and its value is equal to the square root of the cohesive energy density (CED). When the solubility parameter difference between the two substances is less than 4.1 (J/cm^3^)^1/2^, indicating that the compatibility between the two substances is good, the formed system is relatively stable. In this paper, the solubility parameter is used to verify the rationality of the asphalt molecular model. The specific values are shown in Table 7. By comparing the density of the PU-modified asphalt after dynamic simulation with the measured density, the rationality of the PU-modified asphalt model can be verified. The specific values are shown in Table 8.

Obviously, the maximum error of the solubility parameters between the 12 asphalt molecules is 0.808 (J/cm^3^)^1/2^, which is less than 4.1 (J/cm^3^)^1/2^, so the asphalt model is reasonable. By comparing the density of the PU-modified asphalt model obtained from the simulation and the test, it can be seen that the maximum error between the two is 5.8%, and the error is small. Therefore, the asphalt and the PU-modified asphalt model obtained by simulation meet the requirements.

#### 2.3.4. SCA Molecular Model

**Figure 6:**
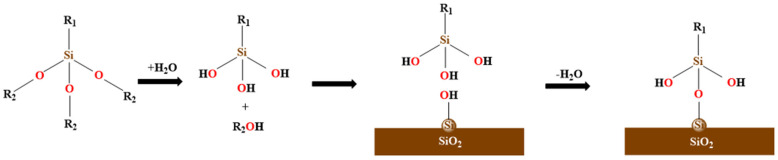
The schematic of chemical reactions for SCA grafting on the SiO_2_ surface.

The SCA is a surface modifier, and its modification on the surface of SiO_2_ belongs to chemical modification; the specific modification process is shown in Figure 6. The hydrophilic end of the SCA is hydrolyzed to form a hydroxyl (-OH), which is further dehydrated and condensed with the hydroxyl on the SiO_2_ surface to form a stable chemical bond. The other end interacts and entangles with the asphalt molecules through intermolecular forces, resulting in physical adsorption that enhances the adhesive ability between asphalt molecules and SiO_2_ [25]. Some SCAs with strong active functional groups can react with the active ingredient in the PU-modified asphalt to form new chemical bonds, which further increases interface strength.

Seven SCA models are used in the simulation process, four of which are KH-550, KH-172, KH-570, and ND-42, respectively, and the specific parameters are shown in Table 9. Since the type of modifier is PU, some excess diisocyanate is often added in the process of use to ensure a sufficient reaction, and there are often residual situations in the process of reacting with diol, in which -OCN reacts with the -NH_2_ of KH-550 to extend the chain length of the SCA. KH-550 was elongated with 2,4-TDI and EG. With DPs of 1, 2, and 3, its lengths after grafting on the SiO_2_ surface were 21.5 Å, 34.2 Å, and 46.2 Å.

#### 2.3.5. Establishment of an Aggregate Model with Grafting SCAs and a Layer Model

The main component of the aggregate is SiO_2_, so this paper uses the SiO_2_ crystal model as the microscopic model of the aggregate. The crystal model was imported through the built-in model library of Materials Studio 2019, and the (1–10) crystal plane was calculated to have the highest activity, so this plane was cut and expanded to the corresponding size as the contact surface with asphalt. The hydrolyzed SCA was grafted to the surface of the SiO_2_ unit cell, and the number of grafts was selected to be 4.

**Figure 7:**
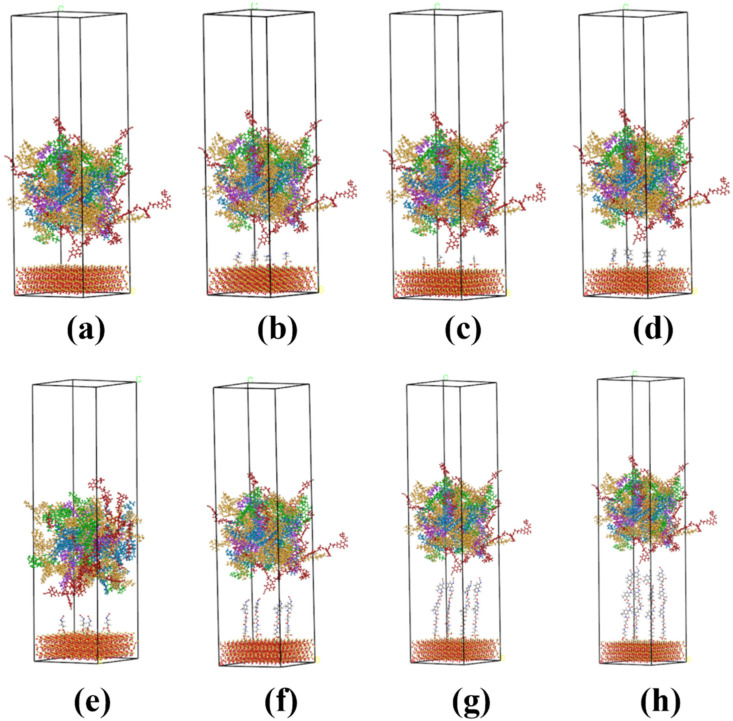
Layer model: (**a**) A_0_; (**b**) B_1_; (**c**) B_2_; (**d**) B_3_; (**e**) B_4_; (**f**) C_1_; (**g**) C_2_; (**h**) C_3_.

The layer model was established by the Build Layer tool; the aggregate model with the SCA was used as Layer 1, the PU-modified asphalt model with a PU content of 20.8% was used as Layer 2, and a 50Å vacuum layer was added to it to eliminate the effects of periodic structures. Taking the layer models without the SCA as the control group A_0_ (as shown in Figure 7a), the layer models with KH-550, KH-172, KH-570, and ND-42 were named B_1_-B_4_ (as shown in Figure 7b–e); the layer models of grafting 2,4-TDI and EG molecules based on KH-550 were defined as C_1_, C_2_, and C_3_ according to the DP and were equal to 1, 2, and 3 (as shown in Figure 7f–h). 

### 2.4. Molecular Dynamics Calculation

The initial density of the asphalt model was set to 1.0 g/cm^3^, and the structure was first subjected to 5000 steps of geometric optimization to reduce the energy of the system. Then, 15 annealing cycles were performed under the NVT ensemble to make the molecule cross the higher potential barrier. Next, find the molecule with the lowest energy configuration. The temperature range is 300–800 K. In the output annealing trajectory file, the structure with the lowest energy is selected as the basis for subsequent molecular dynamics calculations. The annealed model is subjected to 100 ps molecular dynamics calculations under the NPT ensemble to obtain the equilibrium state. The geometric optimization and annealing steps of the layer model are the same as the above steps, and the NVT ensemble is selected in the process of dynamic simulation. During the process, the temperature is set to 130 °C, the temperature control method adopts the Nose method, and the pressure control method adopts the Berendsen method.

#### 2.4.1. Micro-Viscosity

Asphalt viscosity is a key index of asphalt properties, and there are various methods to test and evaluate it in macro tests, but these methods are mostly suitable for asphalt and emulsified asphalt, and they have poor applicability for modified asphalt with high viscosity and prone to segregation. The viscosity of modified asphalt can be evaluated by micro-viscosity. Therefore, this paper uses the shear function of the Forcite module to perform 10^5^ equal-volume shears on the modified asphalt model after dynamics calculations. The ensemble selected NVT, the shear rate was 0.1 ps, and the temperature was set to 130 °C.

#### 2.4.2. Radial Distribution Function

Taking a particle as the central particle, the probability of the existence of other particles at a distance of r is the radial distribution function (RDF), which represents the occupation probability of two particles in each other’s space. In this paper, the RDF is used to evaluate the self-aggregation of PU molecules in PU-modified asphalt. The principle is shown in Equation (5) as follows:

(5) $$g\left(r\right)=\frac{1}{\rho 4\pi r^{2}}\frac{dN}{dr}$$

where ρ is the density of the system, N is the total number of molecules in the system, and r is the distance between the two particles.

#### 2.4.3. Adhesion Works at the Interface

Microscopically, the adhesive performance between interfaces is evaluated by the adhesion work, and the adhesion work is defined as the energy required to separate the asphalt from the aggregate [14]. The specific method is shown in Formula (6) as follows:

(6) $$E_{adhesion}=-\Delta E_{inter}=-\left[E_{total}-\left(E_{asphalt}+E_{aggregate}\right)\right]$$

where $E_{adhesion}$ is the adhesion work between asphalt and aggregate (kcal/mol); $E_{asphalt}$, $E_{aggregate}$, and $E_{total}$ are the energy of the single asphalt, single aggregate, and total system, respectively (kcal/mol); and $E_{inter}$ is the interaction energy between the asphalt and the aggregate (kcal/mol).

#### 2.4.4. Depth of Interaction at the Interface

The adhesion depth of the asphalt on the surface of the aggregate can be indicated by the interactive thickness between the asphalt and the aggregate. The calculation method of the adhesive depth is as shown in Equation (7) as follows:

(7) $$D_{Layer}=D_{end}-D_{begin}$$

In the formula, $D_{layer}$ is the adhesive depth and $D_{begin}$ and $D_{end}$ are the start and end distances of the overlapping regions of the two-phase substances, respectively.

#### 2.4.5. Mean Square Displacement

Mean square displacement (MSD) represents the average value of the square of particle displacement, which can be used to evaluate the movement ability of particles. The specific calculation formula is shown in Equation (8) as follows:

(8) $$MSD=\langle {\left|r_{i}\left(t\right)-r_{i}\left(0\right)\right|}^{2}\rangle$$

In the formula, $r_{i}\left(0\right)$ and $r_{i}\left(t\right)$ represent the position of atom i at the initial time and t time, respectively, and 〈 〉 means taking the average value.

The MSD value is used to represent the intensity of the molecular motion of the PU-modified asphalt, which indirectly verifies the ability of the SCA to bind it. The larger the MSD value, the faster the molecular motion in the system and the worse the mechanical performance and thermal stability.

## 3. Experiment

The asphalt mixture adopts AC-16 gradation; its synthetic gradation is shown in Table 10, and the oil-to-stone ratio is 4.7.

**Figure 8:**
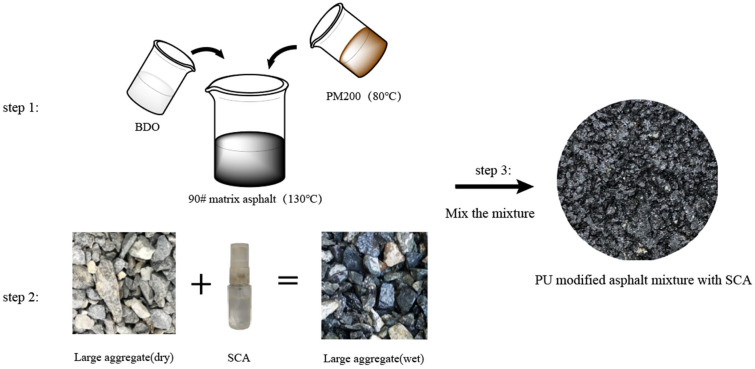
Flowchart of asphalt mixture preparation.

The preparation process of the asphalt mixture composed of PU-modified asphalt and aggregate grafted with the SCA is shown in Figure 8. The preparation of PU-modified asphalt is as follows: heat the asphalt to 130 °C, add 5% BDO (internally mixed), and shear it for 10 min. Then, add 15% PM200 (internally mixed), heat to 80 °C in advance, and shear for 30 min. The prepared PU-modified asphalt was developed in an oven at 130 °C for 60 min. Aggregate preparation includes nSpray 1% KH-550 or 1.12% KH-570 (relative to aggregate mass) of the SCA on the surface of large particle aggregates between 4.75 and 16 mm.

The high- and low-temperature performance of the modified asphalt mixture were evaluated by the high-temperature rutting test and the low-temperature bending test, respectively. The strength of the modified asphalt mixture was evaluated by a Marshall test. The water stability of the modified asphalt mixture was evaluated by the residual stability of the immersion Marshall test and the residual strength ratio of the freeze–thaw splitting test.

## 4. Results and Discussion

### 4.1. Selection of PU Content

**Figure 9:**
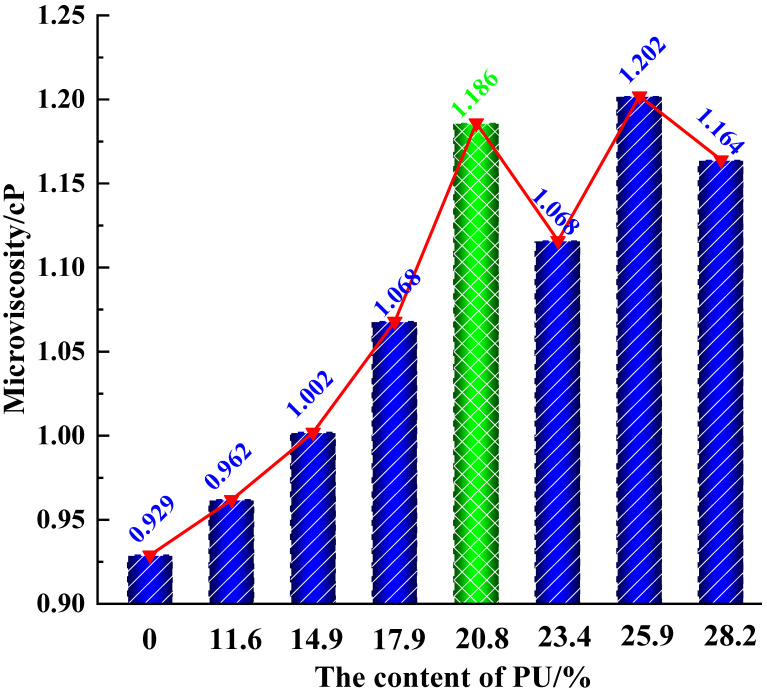
Micro-viscosity of the PU-modified asphalt with different contents.

Figure 9 presents the relationship curve between PU content and the micro-viscosity of PU-modified asphalt. As the PU dosage increases, the micro-viscosity of the modified asphalt gradually rises, but when the dosage reaches 7, the micro-viscosity no longer increases steadily and instead exhibits fluctuations. Within the PU content range of 0–20.8%, the micro-viscosity of PU-modified asphalt demonstrates a near-exponential relationship with PU dosage, where the viscosity increases with higher PU content, indicating polyurethane’s enhancing effect on asphalt’s viscous modification. When PU content exceeds 20.8%, the modified asphalt’s viscosity performance first decreases, then increases, and subsequently decreases again, suggesting that the enhancing effect of polyurethane on asphalt viscosity diminishes at higher dosages and excessive PU content fails to yield better performance. Comparative analysis reveals that the optimal PU dosage for viscosity modification occurs around 20.8%, where the most effective improvement in asphalt viscosity is achieved. As shown by the green bars in Figure 9.

**Figure 10:**
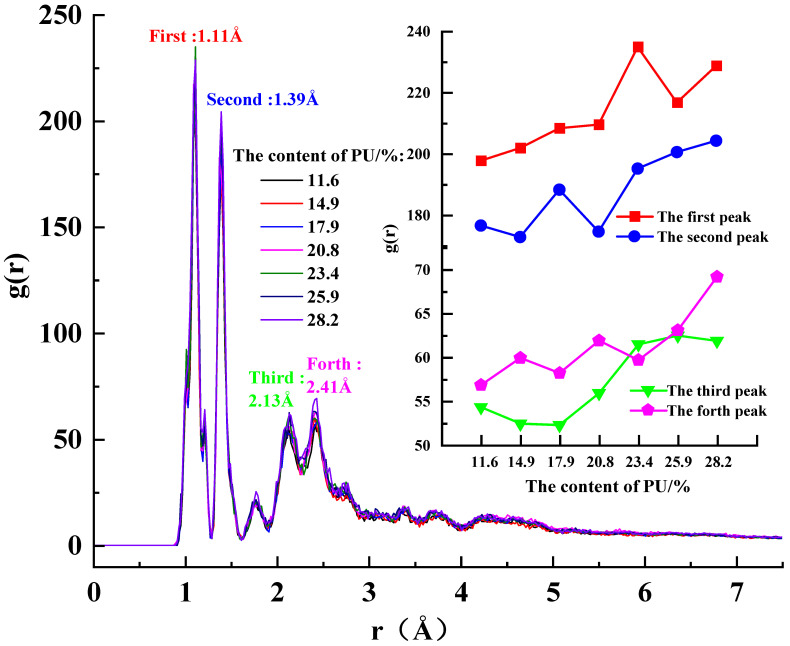
RDF curves of PU in modified asphalt with different contents of PU.

Based on the radial distribution function (RDF) analysis of PU-modified asphalt with different dosages shown in Figure 10, it is found that when the PU content is below 6, the four peaks in the RDF increase uniformly. However, when the PU modifier content reaches 7 or higher, these peaks exhibit significantly faster growth rates. This phenomenon suggests that PU molecules may begin to self-aggregate at this dosage level, disrupting the previously formed network structure and creating inhomogeneity within the PU-modified asphalt system. Consequently, the compatibility between PU and asphalt deteriorates, ultimately leading to instability in micro-viscosity.

Building on these findings, this study determines through micro-viscosity measurements and RDF microstructural analysis that the optimal PU modifier dosage for asphalt modification is 6 (corresponding to an internal incorporation ratio of 20.8%). At this specific dosage, PU-modified asphalt demonstrates the most favorable modification effects, as evidenced by both microscopic structural characteristics and viscosity performance.

### 4.2. Analysis of the Interface Properties

#### 4.2.1. Adhesion Work

**Figure 11:**
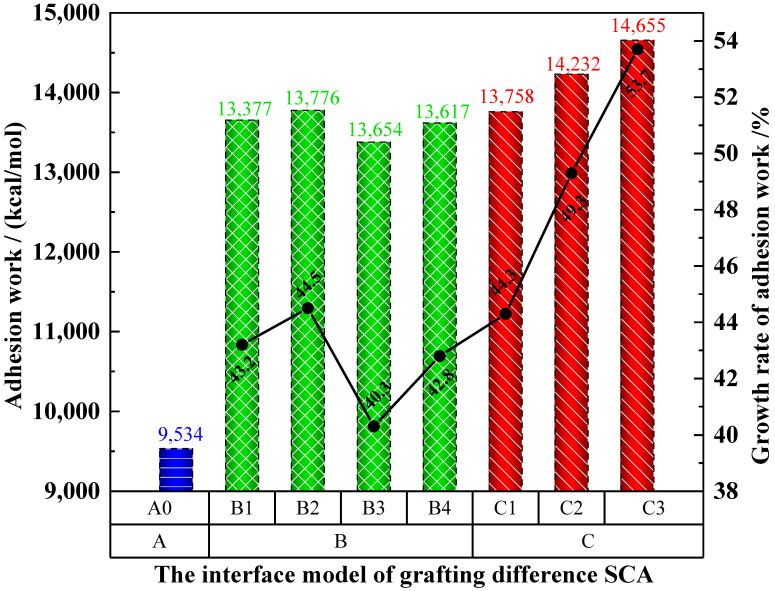
Adhesion work.

Figure 11 shows the Adhesion work of different specimens. A_0_ is the control group, indicated by the blue bar, B_1_ to B_4_ are the experimental groups without chain extension, indicated by the green bar, and C_1_ to C_3_ are the experimental groups with extended chains, indicated by the red bar. The adhesion work between PU-modified asphalt and SiO_2_ grafted with SCAs is higher than that between PU-modified asphalt and pure SiO_2_, which proves that SCAs can improve the interface effect, but the improvement effect is different. Compared with the adhesion work of A_0_, the adhesion work of B_1_-B_4_ increased by 40.3%, 44.5%, 46.8%, and 43.2%, and the adhesion work of C_1_–C_3_ increased by 51%, 51.4%, and 70.2%. Since group C extends the chain length on the basis of B_1_, it can be known that the chain length is one of the important factors affecting the adhesion work. As the chain length of the SCA increases, its adhesion work can grow. Compared with other types of SCAs, in the PU-modified asphalt mixture, -NH_2_ at the end of KH-550 can chemically react with the molecules with -OCN in the PU-modified asphalt to extend the chain length of the SCA and finally enhance the interaction between PU-modified asphalt and SiO_2_. Comparing the adhesion work of B_2_ and B_4_, it can be seen that the shape of the chain and the type of functional groups are also important factors. Functional groups containing heteroatoms can produce non-bonding energy interactions with PU-modified asphalt molecules, which is more than the enhancement of adhesive performance. The length of B_4_ containing heteroatom N is longer than that of B_2_, but compared with the benzene ring with larger molecular weight at the end of B_4_, the overall shape of B_2_ is lighter and easier to embed in PU-modified asphalt to generate adsorption, so B_2_ can better improve the interface adhesion work. Comprehensive analysis shows that the molecular chain length of SCAs, the functional groups, and the shape of the molecular chain are all factors that affect its interface strength. Therefore, when selecting the type of SCA, the physical adsorption performance and reactivity of the SCA should be comprehensively considered.

#### 4.2.2. Adhesion Depth

**Figure 12:**
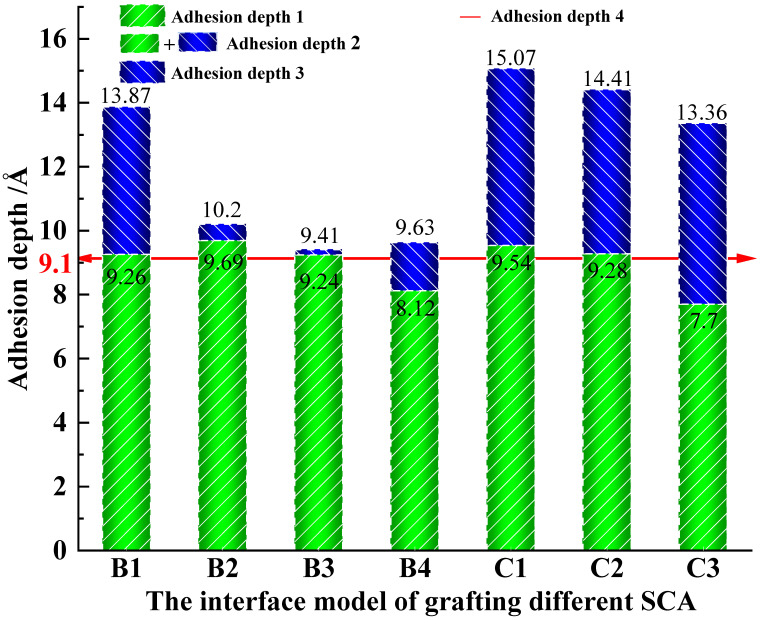
Adhesion depth.

The SCA grafted on the SiO_2_ surface interacted with the PU-modified asphalt molecules, which shortened the distance between the two, enhanced the adhesion ability of the PU-modified asphalt on the surface of SiO_2_, and reduced the possibility of interface damage. Due to the different molecular weights of different types of SCAs, the thickness increase after grafting SCAs on the SiO_2_ surface is different. Therefore, the method of deleting the SCA molecule in the model after dynamic simulation is adopted to obtain the adhesion depth between SiO_2_ and PU-modified asphalt, defined as adhesion depth 1. Adhesion depth 1 is indicated by the green bar. The adhesion depth of the interface containing SCAs is defined as adhesion depth 2, and the difference between the adhesion depth 2 and the adhesion depth 1 is the embedded depth of SCAs in the PU-modified asphalt, which is defined as adhesion depth 3. Depth of adhesion 3 is indicated by the blue bar. The adhesion depth of A_0_ is defined as adhesion depth 4, and the specific results are shown in Figure 12.

From the results of adhesion depth 1, it can be seen that most of the SCA shortens the distance between SiO_2_ and PU-modified asphalt during the interaction process, but for B_4_ and C_3_, there is a decrease relative to adhesion depth 4, which is related to the properties of the two SCAs. Compared with other SCAs in group B, although B_3_ is longer in length, the benzene ring at its end prevents it from entering the voids of PU-modified asphalt and gradually approaches the surface of SiO_2_ during the interaction process. Further contact between SiO_2_ and PU-modified asphalt is hindered, so adhesion depth 1 is reduced. In group C, the interface between C_1_ and C_2_ relies on the strong non-bonding energy interaction between SCAs and PU-modified asphalt to enhance adhesion depth 1, and adhesion depth 3 is enhanced by embedding deeper PU-modified asphalt molecules. However, with the growth of SCAs, its flexibility increases. During the interaction process, an oil film covering the surface of SiO_2_ is formed due to its own agglomeration ability, which hinders the adhesion between SiO_2_ and PU-modified asphalt. In C_3_, adhesion depth 1 is less than adhesion depth 4. It can be seen that suitable SCAs can effectively increase the adhesion depth between asphalt and SiO_2_, reduce the possibility of water and other unfavorable factors intruding from the interface, and improve the interface strength, damage resistance, and aging resistance.

### 4.3. Thermal Stability of the Interface

**Figure 13:**
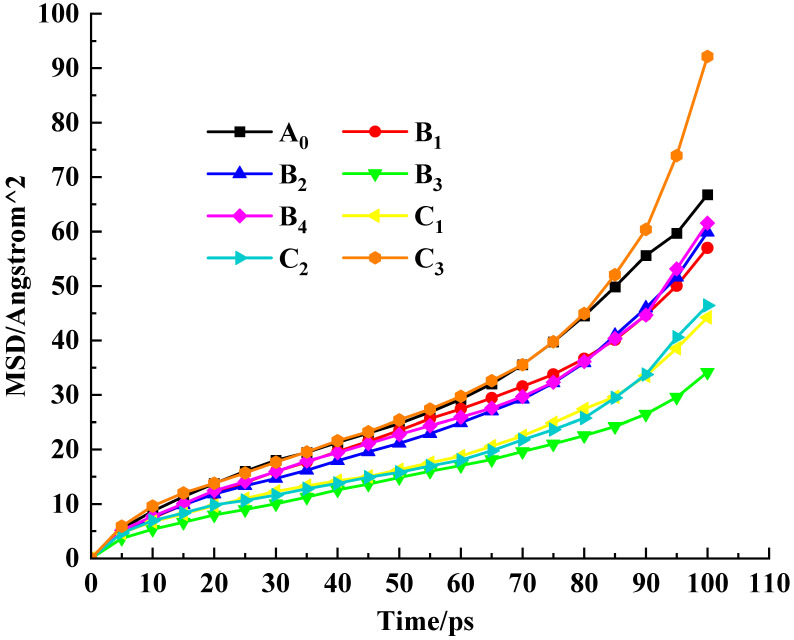
MSD curve of PU-modified asphalt molecules.

At the same time, the thermal stability of the interface at this temperature can be judged by comparing the MSD values of the PU-modified asphalt molecules during the dynamics simulation. As shown in Figure 13, the MSD values of group B all decreased compared to A_0_, which proved the positive effect of SCAs in preventing the thermal motion of PU-modified asphalt molecules, among which B_3_ had the best effect. In group C, with the increase in DP value, the binding ability of SCAs to the movement of the PU-modified asphalt model first increases and then decreases. When the value of DP is 3, the movement ability of PU-modified asphalt molecules is stronger than that without constraint. In C_3_, the molecular weight of SCAs is relatively large, except for the part interspersed into the PU-modified asphalt molecules; the remaining SCAs form an organic molecular interlayer on the surface of SiO_2_. The amorphous state of SCAs also provides a less constrained active platform for PU-modified asphalt molecules, so the thermal stability at the interface of C_3_ is poor. The study reveals that the mechanism of silane coupling agents enhancing the asphalt–aggregate interface can be summarized as follows. After hydrolysis, the silane coupling agent molecules undergo condensation reactions with the aggregate surface, forming stable Si-O-Si covalent bonds at the aggregate end. Meanwhile, at the modified asphalt end, these molecules interact with asphaltene and resin molecules in the asphalt through intermolecular forces. This dual interaction establishes a “molecular bridge” at the asphalt–aggregate interface, thereby significantly improving interfacial strength.

### 4.4. Basic Test Performance Analysis

In accordance with the “Test Methods of Bitumen and Bituminous Mixtures for Highway Engineering” (JTG E20-2011), we conducted standardized tests on the fundamental performance indicators of polyurethane-modified asphalt with incorporation rates of 5%, 10%, 15%, 20%, and 25%. The corresponding test results are presented in Table 11. From Table 11, it can be observed that the addition of polyurethane significantly improves the fundamental performance indicators of the asphalt. As the polyurethane content increases, the penetration of the modified asphalt decreases notably, while the ductility, softening point, and dynamic viscosity increase substantially. This demonstrates that increasing the polyurethane content effectively enhances both the high-temperature stability and low-temperature crack resistance of the modified asphalt. However, it also slightly reduces workability during mixing. When the polyurethane content reaches 20%, the performance improvement reaches its peak. Beyond 25%, further increases in polyurethane content do not lead to significant additional performance gains.

Taking the hot mix SBS-modified asphalt mixture D as the control group, the road properties of the warm PU-modified asphalt mixture E were evaluated. Comparing the pavement properties of warm mix PU-modified asphalt mixtures F and G, which contain KH-550 and KH-570, with E, the enhancement effect of different SCAs on the interface is obtained. The specific composition of each group of mixtures is shown in Table 12. The test results are evaluated according to the Technical Specification for Highway Asphalt Pavement Construction (JTG F40-2004). The basic performance indexes of the four groups of asphalt mixtures are shown in Table 12.

Table 13 shows the high- and low-temperature performance of the asphalt mixture. The stability of the rut indicates the ability of asphalt to resist rutting deformation under high-temperature conditions, and the four groups of mixtures all meet the specification requirements. Comparing D and E, the high-temperature performance of the warm mix PU-modified asphalt mixture is better than that of the hot mix SBS-modified asphalt mixture. Comparing E with F and G, the addition of SCAs can improve the high-temperature performance of the warm mix PU-modified asphalt mixture, but the effect is not obvious. The low-temperature bending test results show the low-temperature crack resistance of the asphalt mixture. The temperature is set to -10 °C, and the loading rate is selected to be 50 mm/min. The flexural tensile strains of the four kinds of mixtures all meet the specification requirements. Comparing the flexural tensile strength values, the flexural tensile strength of the warm mix PU-modified asphalt mixture is lower than that of the hot mix SBS-modified asphalt mixture, but it is improved after adding SCAs to the aggregate, and KH-550 is better than KH-570.

The overall strength and elastic toughness of the mixture can be evaluated by the Marshall stability and flow value. It can be seen from Table 13 that the Marshall stability and flow value of the four groups of mixtures meet the requirements of the specification. Comparing D and E, the strength of the warm mix PU-modified asphalt mixture is greater than that of the hot mix SBS-modified asphalt mixture. Comparing E with F and G, the SCA can enhance the strength of the PU-modified asphalt mixture, and the effect of KH-550 is better than that of KH-570. Combined with the analysis of the adhesion work of B_1_ and B_3_ in the molecular simulation, it is obtained that -NH_2_ of KH-550 can chemically react with the molecules with -OCN in PU-modified asphalt to extend the chain length, which can obtain stronger interfacial force and improve the overall strength of the mixture.

It can be seen from Table 13 that four groups of residual stability of the immersion Marshall test all meet the requirements of the specification. Comparing D and E, it can be seen that the water damage resistance of the hot mix SBS-modified asphalt mixture is better than that of the warm mix PU-modified asphalt mixture. Comparing E with F and G, it can be seen that the addition of the SCA enhances the water damage resistance of the PU-modified asphalt mixture. In the residual strength ratio test of the freeze–thaw splitting test, except for group E, all meet the specification requirements, and the value of group D is greater than that of groups F and G. This shows that the water damage resistance of the warm mix PU-modified asphalt mixture without the SCA still has certain defects, but it can meet the requirements of the specification by adding the SCA to the aggregate, and KH-550 works better than KH-570.

## 5. Conclusions

This paper starts with two aspects of improving asphalt properties and asphalt–aggregate interface performance. Through the molecular simulation method, the optimal content of PU and the SCA type with the best effect on improving the interface strength are selected. Through the macro test used to verify the reinforcement effect of the selected material under warm mixing temperatures, the following conclusions are finally obtained:

(1) At the microscopic level, the micro-viscosity of PU-modified asphalt with different contents and the RDF of PU are analyzed, and it can be seen that when the PU content is 20.8%, the compatibility between PU and asphalt and the micro-viscosity of PU-modified asphalt are the best.
(2) The adhesion work, adhesion depth, and thermal stability between PU-modified asphalt and SiO_2_ grafted with different SCAs were investigated by molecular simulation. It can be seen that the chain length and shape of the SCA, and whether the functional group can react with PU, are important influencing factors. The enhancement effect of KH-570 on the interface is greater than that of KH-550, but due to the reactivity of the amino at the end of KH-550, it is predicted that the interface enhancement effect of KH-550 is the best in the actual application.
(3) Taking the hot mix SBS-modified asphalt mixture as the control group, the properties of the warm mix PU-modified asphalt mixture with or without the SCA were analyzed. The high-temperature and strength performance of the warm mix PU-modified asphalt mixture are better than those of the hot mix SBS-modified asphalt mixture, but the low-temperature performance is slightly lacking, and the water damage resistance cannot meet the requirements of the specification. The two performances of the PU-modified asphalt mixture after adding SCAs on the aggregate are enhanced, and both meet the requirements of the specification.
(4) Comparing the basic performance of PU-modified asphalt with and without SCAs by the macro test, it can be seen that the improvement effect of KH-550 is stronger than that of KH-570. Combined with the conclusion of the molecular simulation, it is concluded that KH-550 chemically reacts with molecules with the isocyanate in PU-modified asphalt, which effectively prolongs the chain length of SCAs and forms a larger cross-linked network between asphalt and aggregate to provide better performance for the mixture. So, KH-550 has better performance in practical applications.

## Figures and Tables

**Table 1:** Basic performance indexes of asphalt.

Asphalt	25 °C Penetration/0.1 mm	15 °C Ductility/cm	Softening Point/°C
CNOOC 90#	92.8	107	46.9

**Table 2:** Basic performance indexes of Wanhua PM200 polymerized isocyanate.

25 °C Physical State	Viscosity/(25 °C, MPa·s)	NCO Mass Fraction/%	Density/(25 °C, g·cm^−3^)
Brown liquid	150~250	30.2~32.0	1.22~1.25

**Table 3:** Basic performance indexes of 1,4-butanediol.

25 °C Physical State	Viscosity/(25 °C, MPa·s)	Density/(25 °C, g·cm^−3^)	Content/%
Colorless oily liquid	72	1.02	≥99

**Table 4:** Basic performance indexes of SCAs.

Full Chemical Name	Abbreviated Name	25 °C Physical State	Density/(25 °C, g·cm^−3^)
(3-Aminopropyl) triethoxysilane	KH-550	colorless liquid	0.95
(Trimethoxysilyl) propyl methacrylate	KH-570	light yellow liquid	1.05

**Table 5:** Basic performance indexes of SBS.

Physical State	Proportion	Tensile Strength/0.1 mm	Hardness	Elongation at Break/%
White particles	0.93	120	83	345

**Table 6:** Molecular composition of the asphalt model.

Asphalt Component		Number of Molecules	Mass Fraction/%
Asphaltene	Asphaltene-phenol	3	13.3
	Asphaltene-pyrrole	3	
	Asphaltene-thiophene	2	
Polar Aromatics	Pyridinohopane	4	31.1
	Thin-isorenieratane	7	
	Benzobisbenzothiophene	6	
	Quinolinohopane	4	
	Trimethylbenzene-oxane	7	
Naphthene Aromatics	PHPN	17	37.6
	DOCHN	18	
Saturates	Squalene	7	18.0
	Hopane	8	

**Table 7:** CED and solubility parameters of 12 asphalt molecules.

Asphalt Component		Cohesive Energy Density/(×108 J/m^3^)	Solubility Parameter /(J/cm^3^)^1/2^
Asphaltene	Asphaltene-phenol	2.78	16.685
	Asphaltene-pyrrole	2.73	16.524
	Asphaltene-thiophene	2.64	16.265
Polar Aromatics	Pyridinohopane	2.66	16.323
	Thin-isorenieratane	2.79	16.724
	Benzobisbenzothiophene	2.86	16.934
	Quinolinohopane	2.71	16.47
	Trimethylbenzene-oxane	2.73	16.55
Naphthene Aromatics	PHPN	2.80	16.761
	DOCHN	2.76	16.624
Saturates	Squalene	2.60	16.142
	Hopane	2.60	16.126
Maximum Difference Value		0.16	0.808

**Table 8:** Model density and measured density of asphalt and PU-modified asphalt.

Number of PU Molecules	PU Content/%	Model Density/(g/cm^3^)	Measured Density/(g/cm^3^)	Error /%
0	0	0.994	1.02	2.6
3	10.3	1.007	1.03	2.3
4	13.3	1.014	1.04	2.6
5	16	1.016	1.04	2.3
6	18.7	1.017	1.04	2.3
7	21.1	1.02	1.05	2.9
8	23.5	1.022	1.08	5.6
9	2	1.03	1.09	5.8

**Table 9:** SCA descriptions.

No.	Full Chemical Name	Abbreviated Name	Chemical Structure	Relative Molecular Mass	Length After Grafting/Å
1	(3-Aminopropyl) triethoxysilane	KH-550	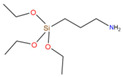	221	7.353
2	Vinyltris (b-methoxyethoxy) silane	KH-172	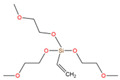	280	7.090
3	(Trimethoxysilyl) propyl methacrylate	KH-570	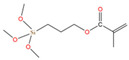	248	10.71
4	Aniline methyl triethoxy silane	ND-42	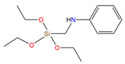	269	11.37

**Table 10:** AC-16 synthetic grading.

	Mass Percentage Through Mesh (mm)/%										
Sieve size/mm	19	16	13.2	9.5	4.75	2.36	1.18	0.6	0.3	0.15	0.075
Passing rate/%	100	93.6	86.3	74.1	52.7	32.4	24.1	15.6	9.4	6.1	5.4

**Table 11:** The fundamental performance of polyurethane-modified asphalt.

PU Content /%	25 °C Penetration/0.1 mm	15 °C Ductility/cm	Softening Point/°C	60 °C Dynamic Viscosity/(Pa.s)
5	92.8	108	46.9	162
10	70.5	116	60.3	175
15	62.3	125	78.9	268
20	50.2	138	85.6	345
25	45.3	138	86.1	351

**Table 12:** Composition of mixtures.

No.	Modifier	Modifier Content/%	Aggregate Surface Treatment
D	SBS	5%	/
E	PU	20%	
F			KH-550 (1%)
G			KH-570 (1.12%)

**Table 13:** Basic performance indexes of modified asphalt mixtures.

Performance Indexes of Asphalt Mixtures		Unit	Modified Asphalt Mixtures					Specification Requirements
			D	E	F	G	Maximum Error/%	
High-temperature performance	Stability of the rut	times/mm	4526	6172	6285	6192	4.5	≥2400
Low-temperature performance	Flexural tensile strength	MPa	9.2	8.29	8.65	8.46	3.6	/
	Flexural tensile strain	με	3216	3165	3379	3212	6.3	≥3000
Strength performance	Marshall stability	kN	10.3	11.5	12.8	12.3	2.1	≥8
	Flow value	mm	2.68	2.26	2.16	2.19	4.5	≤4
Water stability	Residual stability of the immersion Marshall test	%	90.3	86.9	89.4	87.8	6.4	≥85
	Residual strength ratio of the freeze–thaw splitting test	%	85.3	78.6	84.7	82.5	5.7	≥80

## Data Availability

Data are contained within the article.

## Abbreviations

The following abbreviations are used in this manuscript:

EG	Ethylene glycol
PU	Polyurethane
RDFs	Radial distribution functions
SCAs	Silane coupling agents
